# Supplementary material for: Assessing processing-based measures of implicit statistical learning: Three serial reaction time experiments do not reveal artificial grammar learning
Source: PLoS One. 2024 Sep 20;19(9):e0308653. doi: 10.1371/journal.pone.0308653 (PMC11414973; doi:10.1371/journal.pone.0308653)
Supplement: S1 File — This file contains all supporting information for this manuscript including: S1 Table: duration of breaks taken during experiment; S2 Table: descriptive statistics; S1 Fig: analyses of reaction times based on X element (RTX-B); S2 Fig: reaction times for each element (A, X and B) for all experiments; S3 Fig: methods for Experiment 3, adjacent condition. (DOCX) [file pone.0308653.s001.docx]

**Supplementary Materials for**

**Jenkins et al.**

**Assessing Processing-Based Measures of Implicit Statistical Learning: Three Serial Reaction Time Experiments Do Not Reveal Artificial Grammar Learning**

**Supplementary Table 1.** Break durations (in seconds) and their relationship to implicit statistical learning, across all three experiments. In each experiment, participants were offered the opportunity to take a break part way through the experiment, to avoid fatigue or boredom interfering with possible learning effects. We provide descriptive statistics on the duration of these breaks, as well as correlations between break durations and performance on key measures of implicit statistical learning. There was no relationship between break duration and learning.

|  | *Experiment 1* | *Experiment 2* | *Experiment 3*  *(Adjacent)* | *Experiment 3*  *(Nonadjacent)* |
| --- | --- | --- | --- | --- |
| *Mean Break (+/- SEM)* | *27.34 (4.79)* | *49.77 (7.48)* | *11.40 (1.72)* | *13.02 (2.86)* |
| *Minimum Break* | *4.75* | *5.47* | *5.12* | *1.39* |
| *Maximum Break* | *93.60* | *226.13* | *29.78* | *44.90* |
|  |  |  |  |  |
| *Correlation between break duration and learning* |  |  |  |  |
| *RT_A-B_ in Block 6 – Ungrammatical Block* | *r = 0.15*  *p = 0.44* | *r = 0.22*  *p = 0.18* | *-* | *-* |
| *RT_A-B_ in Recovery Block – Ungrammatical Block* | *r = 0.08*  *p = 0.68* | *r = 0.18*  *p = 0.27* | *-* | *-* |
|  |  |  |  |  |
| *(Block 5 low frequency - high frequency RT_A-B_) - (Block 4 low frequency -high frequency RT_A-B_)* | *-* | *-* | *r = -0.21*  *p = 0.42* | *r = 0.07*  *p = 0.80* |

**Supplementary Table 2.** Descriptive statistics for mean reaction times (RT_A-B_, in ms) for all blocks of all experiments.

|  |  | Mean Reaction Time Differences (RT_A-B,_ ms) | | | | | | | |
| --- | --- | --- | --- | --- | --- | --- | --- | --- | --- |
| Experiment 1 |  | Block 1 | Block 2 | Block 3 | Block 4 | Block 5 | Block 6 | Testing | Recovery |
|  |  | 124.64 | 89.38 | 101.07 | 121.69 | 132.86 | 121.66 | 74.76 | 108.16 |
|  |  |  |  |  |  |  |  |  |  |
| Experiment 2 |  | Block 1 | Block 2 | Block 3 | Block 4 | Block 5 | Block 6 | Testing | Recovery |
|  |  | 58.01 | 44.75 | 65.38 | 71.12 | 94.39 | 126.01 | 88.12 | 113.80 |
|  |  |  |  |  |  |  |  |  |  |
| Experiment 3 | | Block 1 | Block 2 | Block 3 | Block 4 | Block 5 | Block 6 | Block 7 | Block 8 |
| Adjacent | Grammatical | -38.88 | -41.95 | -30.45 | -9.51 | -27.41 | -27.31 | -30.82 | -55.11 |
|  | Ungrammatical | -98.46 | -19.91 | -38.38 | -60.45 | -4.04 | -44.02 | -35.49 | -4.44 |
| Nonadjacent | Grammatical | -59.83 | -74.13 | -45.45 | -38.31 | -65.30 | -83.53 | -59.04 | -54.09 |
|  | Ungrammatical | 4.29 | -87.47 | -28.39 | -52.84 | -86.41 | -76.02 | -45.47 | -36.64 |


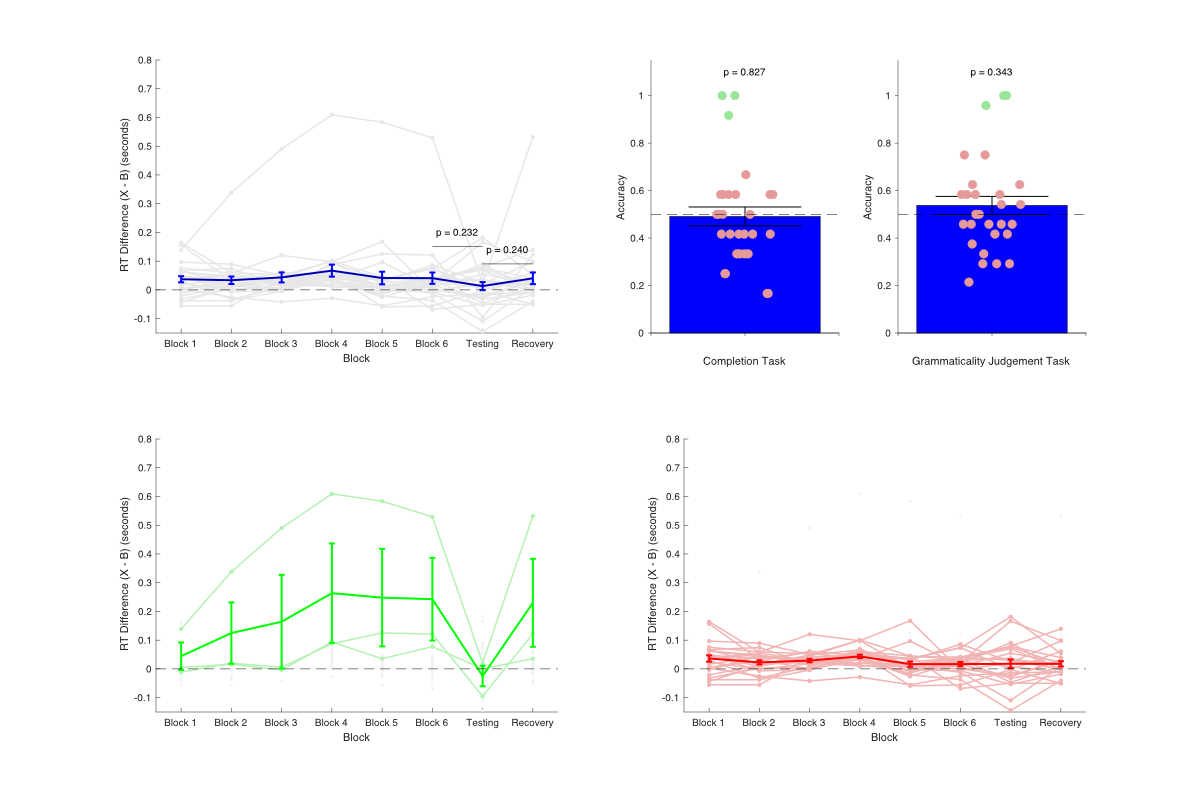


**Supplementary Figure 1.** Data from Experiment 1 reanalyzed using reaction time data calculated based on the ‘X’ elements rather than the ‘A’ elements (RT_X-B_). It was possible that participants’ responses to the initial ‘A’ elements in the sequences may have been noisy, therefore we reanalyzed the data using the central ‘X’ element as the baseline. The results are unchanged, and no significant results were observed at the group level. Note that the results from the Sequence Completion and Grammaticality Judgement Tasks remain unchanged from Fig. 2.


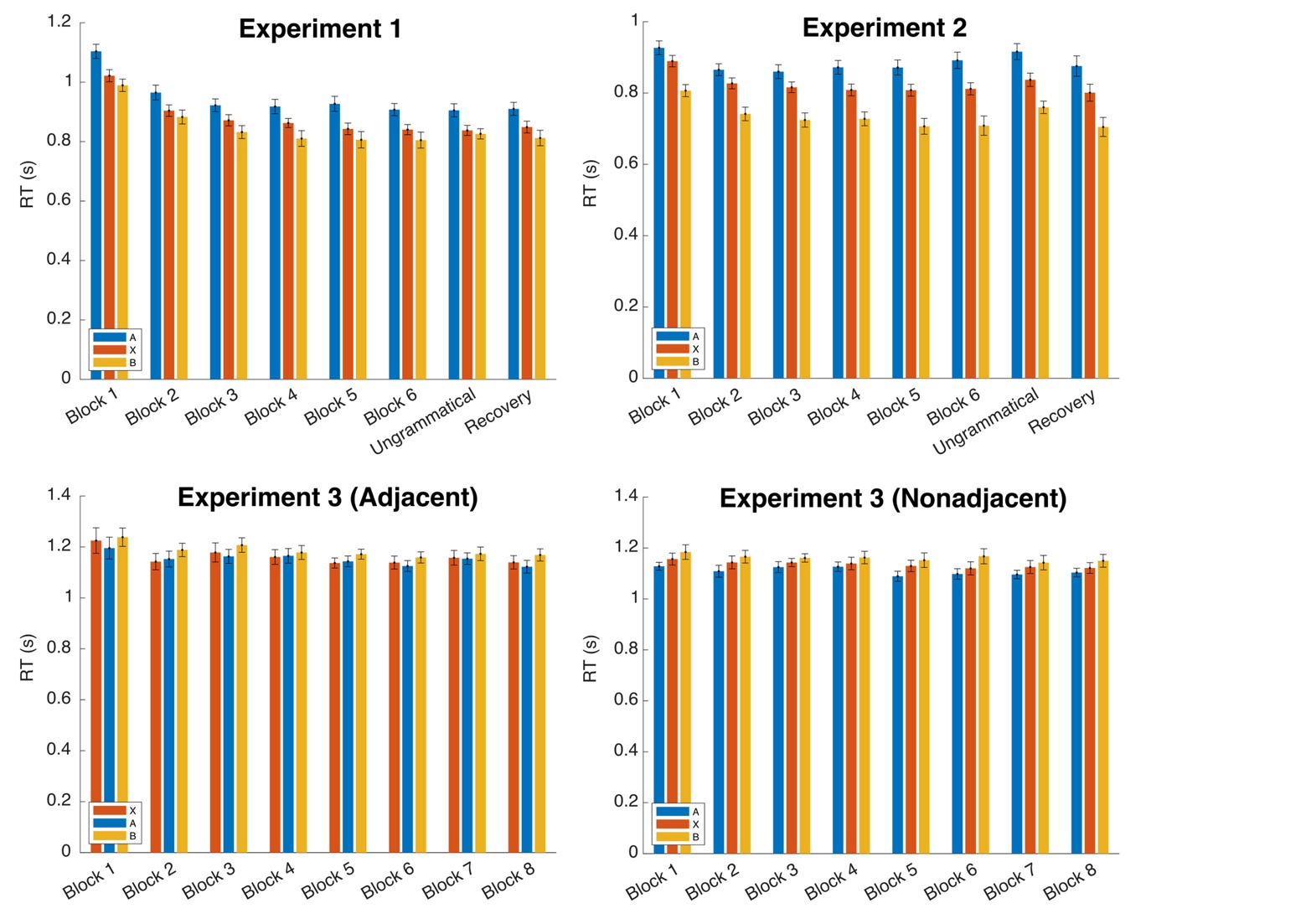


**Supplementary Figure 2.** Reaction times in response to each element in each block, for each experiment. Data show mean (+/-SEM) of the median reaction times (in seconds) for each element, block, and experiment.

**
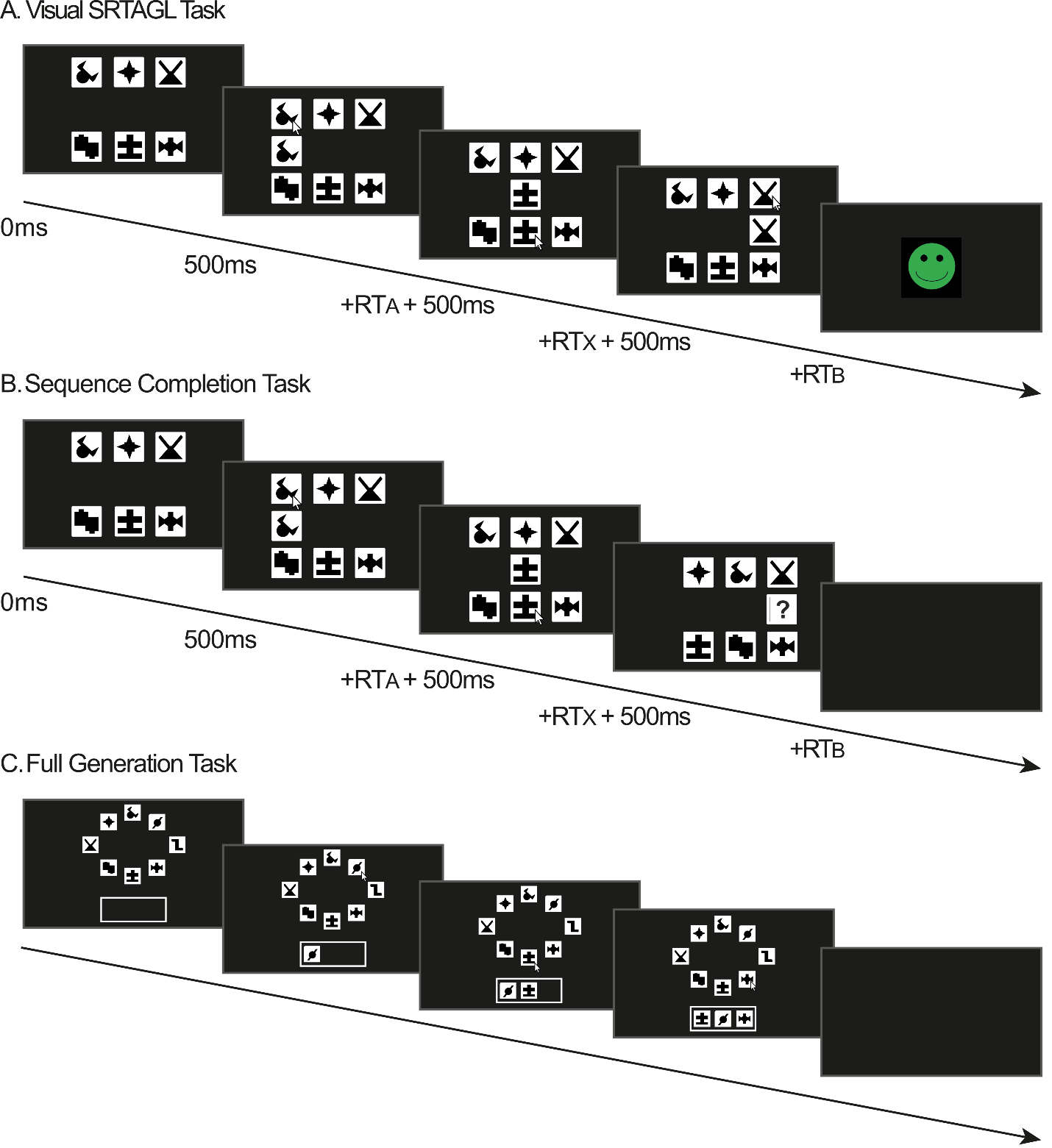
**

**Supplementary Figure 3**. Trial design for the adjacent condition of Experiment 3, to complement Fig. 6.
